# Molecular characterization of transesterification activity of novel lipase family I.1

**DOI:** 10.1042/BSR20220654

**Published:** 2022-10-07

**Authors:** Titin Haryati, Made Puspasari Widhiastuty, Fida Madayanti Warganegara, Akhmaloka Akhmaloka

**Affiliations:** 1 Doctoral Program of Chemistry, Faculty of Mathematics and Natural Science, Institut Teknologi Bandung, Jl. Ganesha 10, Bandung, 40132, Jawa Barat, Indonesia; 2 Indonesia, National Research and Innovation Agency, Gedung B.J. Habibie Jalan M.H. Thamrin Nomor 8, Jakarta Pusat 10340, Indonesia; 3 Biochemistry Research Group, Faculty of Mathematics and Natural Science, Institut Teknologi Bandung, Jl. Ganesha 10, Bandung, 40132, Jawa Barat, Indonesia; 4 Department of Chemistry, Faculty of Science and Computer, Universitas Pertamina, Jl. Teuku Nyak Arief, Jakarta Selatan, Jakarta, 12220, Indonesia

**Keywords:** Lipase recombinant, molecular docking, molecular interactions, Solvent tolerance, Thermostability, Transesterification

## Abstract

Lipase’s thermostability and organic solvent tolerance are two crucial properties that enable it to function as a biocatalyst. The present study examined the characteristics of two recombinant thermostable lipases (Lk2, Lk3) based on transesterification activity. Conversion of C12-C18 methyl ester with paranitrophenol was investigated in various organic solvent. Both lipases exhibited activity on difference carbon chain length (C12 - C18, C18:1, C18:2) of substrates. The activity of Lk2 was higher in each of substrate compared with that of Lk3. Experimental findings showed that the best substrates for Lk2 and Lk3 are C18:1 and C18:2 respectively, in agreement with the computational analysis. The activity of both enzymes prefers on nonpolar solvent. On nonpolar solvent the enzymes are able to keep its native folding shown by the value of radius gyration, solvent–enzyme interaction and orientation of triad catalytic residues. Lk3 appeared to be more thermostable, with maximum activity at 55°C. The presence of Fe^3+^ increased the activity of Lk2 and Lk3. However, the activity of both enzymes were dramatically decreased by the present of Ca^2+^ despite of the enzymes belong to family I.1 lipase known as calcium dependent enzyme. Molecular analysis on His loop of Lk2 and Lk3 on the present of Ca^2+^ showed that there were shifting on the orientation of catalytic triad residues. All the data suggest that Lk2 and Lk3 are novel lipase on the family I.1 and both lipase available as a biocatalyst candidate.

## Introduction

Lipase are a group of hydrolase enzymes showing broad catalytic capabilities, including hydrolysis, esters synthesis, transesterification (acidolysis, interesterification and alcoholysis) and also aminolysis [1,2]. Lipase families were grouped into eight families and six sub-families of true lipase [3]. Today, lipase families comprise 35 families and 11 subfamilies of true lipase [4,5]. Lipases are used in a variety of industries, including agriculture, detergents, food, nutraceuticals and biodiesels [6].

Organic solvents are used in the majority of industrial-scale synthetic processes because of their ability to alter reaction balance toward synthetic direction and ease of product recovery, higher activity and stability, stereoselectivity and regiospecificity. [7]. Therefore, searching for organic solvent tolerance lipase is very important. Tolerance on organic solvents is positively correlated with thermostability [8]. These two characteristics make lipase can be fulfilled as industrial biocatalyst.

Thermostable and solvent tolerance enzymes could be obtained through protein engineering strategies in collaboration with discovery of new lipase [9]. However, protein engineering strategy requires a complete understanding of the structure of lipase using pre-characterized or commercial lipases. Hence, discovery of new lipase with both specific characteristic are important and needed to be explore. The source of a new lipases could be obtained by screening from cultivated microorganisms or metagenome approach [9–13].

Pseudomonas sp. lipase are emerging biocatalysts with thermostable and organic solvent tolerance character. Amano has marketed lipases from *P. cepacia* (Lipase PS) and *P. fluorescens* (Lipase AsK) [14]. The other widely used Pseudomonas lipase is *Pseudomonas stutzeri* lipase which have aminolysis and transesterification activity and also have enantiomer selectivity [15–18]. Studies of metagenomics related to *P. stutzeri* lipase have so far been rare.

This report investigated Lk2 and Lk3 thermostability and characterized transesterification activity in organic solvents. The enzymes are recombinant thermostable lipases isolated by metagenomic approach from domestic compost [19]. Based on homological analysis, the lipases are most closely related to *P. stutzeri* lipase belonging to family I.1. Both lipases have been characterized based on hydrolysis activity [13]. In *Escherichia coli* BL21(DE3), Lk2 and Lk3 are overexpressed and extracted as active soluble enzymes using the thermolysis technique. As the physiological and biochemical properties of Lk2 and Lk3 may differ despite being obtained from the same source, experimental and *in silico* analysis were carried out to probe a better understanding on molecular interactions of lipase–substrate, ion metal, and thermostability.

## Materials and methods

### Chemicals, plasmids and bacterial strains

Lipase genes *LK2* and *LK3* were obtained from our collection. The recombinant plasmid pET-30a(+)-*LK2* and pET-30a(+)-*LK3* were transformed into *E. coli* strain BL21 (DE3) (15). pET-30a (+) plasmids vector were used as an expression vector. All of the chemicals were bought from Merck (Merck, Germany) and Sigma (Sigma, Chemicals, U.S.A.).

### Heterologous expression

LB medium contained kanamycin sulfate (50 g/ml) was used to cultivate *E. coli* BL21 (DE3) with recombinant plasmid and shaken at 150 rpm at 37°C. IPTG was added at a final concentration of 1 mM, and the mixture was incubated at 37°C for 4 h when the 600 nm absorbance was 0.6–0.7. Centrifugation was employed to extract cells, which were then stored at −20°C until needed.

### Membrane cell disruption by thermolysis

By adding 0.1% sodium dodecyl sulfate (SDS), The cells were resuspended in 50 mM sodium phosphate buffer (pH 8.0) and incubated for 30 min at 50°C. After centrifuging the mixture for 30 min at 11900 g, the supernatant was recovered. To avoid denaturation, the supernatant was settled with a 30 mM K_2_HPO_4_ buffer.

### SDS–PAGE and zimography

SDS-PAGE was done at 110 V on a 12% running gel with SDS running buffer. Protein bands were visualized by using 0.1% Commasie Brilliant Blue to stain the gel. The purified enzyme’s molecular mass was calculated using Protein marker III (pre-stained), peqGOLD Protein Ladder. The polyacrylamide gel-embedded lipase was renaturated with 50 ml of sodium phosphate buffer (50 mM, pH 8) containing 0.1% tritone overnight in preparation for the zymographic examination. Subsequently, 1-naphthalene laurate was incubated with lipase in 50 ml sodium phosphate buffer (50 mM, pH 8) at 50°C for 4 h. By adding 25 mg of fast blue dye, the staining activity was performed.

### Purification of recombinant lipase

Ni-NTA agarose matrix (1.5 ml) on column was balanced with 12 ml miliQ water, After that, 15 ml PBS 50 mM at pH of 8 (1% (V/V) Triton-X 100, 100 mM NaCl) were added. The recombinant enzymes were collected on a column after the thermolysis stage. Then, the column washed with PBS 50 mM at pH of 8 contained 100 mM NaCl. The elution buffer was used to elute the bound recombinant enzymes (50 mM PBS pH 8, 300 mM NaCl, imidazole 100 mM). The elution fraction was dialyzed overnight used PBS buffer pH 8 at 4°C to remove any remaining SDS and imidazole. The recombinant enzyme was then concentrated using diafiltration (Merck Millipore). Using the Bradford technique and a bovine serum albumin standard, the amount of protein in the sample was determined [20]. The concentrated enzymes were used for transesterification assay.

### Transesterification activity assay

Transesterification activity of recombinant lipase Lk2 and Lk3 were measured using Pohnlein method with slight modification [21]. Transesterification was performed between methyl esters and para-nitrophenol in organic solvents at various temperature. The conversion of para-nitrophenol into para-nitrophenyl esters was monitored through decreasing absorbance at 400 nm. The reaction procedures as followed: 10 µl concentrated recombinant lipase was mixed with 990 µl organic solvents (contained 4 mM pNP and 24 mM methyl esters). In a 2 ml reaction tube, the reaction was carried out for 10 min at 150 rpm. Enzymes precipitation were done by centrifuged the tube at 11,900 ***g*** for 3 min, then 50 µl of the upper layer was removed and mixed with 1 ml of Tris Cl buffer pH7 (0.1 percent triton). UV-Vis spectrophotometry was used to measure the remaining pNP (Genesis 10S UV-Vis, Thermo Scientific). The amount of enzyme that released one mole of p-nitrophenyl ester in 1 min was defined as one enzymatic unit. All of the tests were carried out in triplicate, including the blanks (without the addition of enzymes).

### Purified recombinant lipase characterization

#### Substrate and solvent specificity determination

Various methyl esters were used to test substrate specificity (varying fatty acids carbon chain length): laurate (C12), myristate (C14), palmitate (C16), stearate (C18), oleate (C18:1) and linoleate (C18:1) (C18:2). Various organic solvents, such as n-hexane, acetone and acetonitrile, were used to test the solvent specificity.

#### Temperature’s effect

Shaking at 150 rpm for 10 min in n-hexane with 4 mM p-nitrophenol and 24 mM methyl palmitate as the substrate at various temperatures (35, 40, 45, 50, 55 and 60°C) determined the optimum temperature for transesterification activity. The pure recombinant lipase was pre-incubated at temperatures (50 and 55°C) for 24 h for stability experiments, with residual activity measured every 2 h. At 0 h, the activity level was recorded as 100%.

#### Determination of kinetic parameters

Bisubstrate kinetics were determined at different concentrations of methyl esters and pNP using n-hexane as a solvent at the optimal temperature of each lipase. The concentrations of pNP ranged from 0.6 to 4 mM and methyl ester from 3 to 24 mM. The *K*_m_ and *V*_max_ values were calculated from the Lineweaver–Burk linear regression plot. The *k*_cat_ values were calculated by dividing the *V*_max_ by the lipase concentration of the reaction mixture.

#### Metal ions effect

After 30 min incubation of pure recombinant lipase in the presence of 5 mM ZnCl_2_, NiCl_2_, FeCl_3_ and CaCl_2_, the residual activities were measured to investigated the influence of metal ions on transesterification activity. The activity obtained in the absence of metal ions was taken to be 100%.

#### Modeling structure and docking

Homology modeling was used to construct 3D protein structures using AlphaFold2 (https://colab.research.google.com/github/sokrypton/ColabFold/blob/main/AlphaFold2.ipynb) [22]. Validation of the resulting structure based on the Ramachandran plot using MolProbity [23]. Protein minimization was not performed to prevent amino acid residues from being present in the dissallowed area on the basis of the Ramachandran plot [24]. PubChem data were used to create the ligand structure (https://pubchem.ncbi.nlm.nih.gov/) [25]. The ligand was then minimized by using Orca [26] and recorded as PDB with the Avogadro software [27]. Flexible docking was performed using Autodock Vina [28]. Three catalytic and two oxyanion holes residues were set as flexible, while enzymes without these five flexible residues were set as rigid receptors. Preparation for docking and grid determination were carried out by Autodock MGLTools-1.5.6 [29]. Polar hydrogen and Kollman charges were added to the enzymes structure, while Gastaiger charges were computed for the ligand. Grid dimension covered conserved region and oxyanion hole. Grid spacing was kept at 1 Å. Docking visualization using Pymol and Ligplot [30,31]. PLIP server is used to analyze protein–ligand interactions (https://plip-tool.biotec.tu-dresden.de/plip-web/plip/index) [32].

#### Substrate binding area

The possible binding pockets for the 3D structure of Lk2 and Lk3 were predicted using Castp’s online server (http://sts.bioe.uic.edu/castp/calculation.html). The CASTp algorithm calculates the size and volume of projected pockets [33].

#### Solvent preference

Solvent preference was confirmed by dynamic molecular simulation using structure minimization in various organic solvents. This is accomplished by defining minimization using the implicit mode (implicit solvent calculations Born generalized parallel with NAMD) [34]. The GBIS model treats polar solvent as a dielectric continuum and screens electrostatic interactions between solute atoms as a result. A total of 40,000 minimization steps were performed with VMD plugin QwikMD [34]. 3D protein structure was dissolved and minimize on various organic solvents (n-hexane, acetone and acetonitrile). The adjustment of the solvent dielectric constant was carried out manually in the configuration file.

#### Structure stability

The SCooP algorithm, a Gibbs–Helmholtz equation-based program that predicts protein stability assuming monomeric proteins and a two-state folding transition, was used to derive temperature-dependent protein stability predictions (http://babylone.ulb.ac.be/SCooP) [35]. Protein stability prediction is also analyzed by calculated hydrophobic cluster, hydrogen bond and salt bridge using ProteinTools website (https://proteintools.uni-bayreuth.de.) [36].

#### Metal ion binding

Binding potential prediction analysis for protein–metal ion were performed by uploaded .pdb file to the Metal Ion Binding servers (http://bioinfo.cmu.edu.tw/MIB/) [37].

## Results and discussions

### Lk2 and Lk3 expression and purification in *E. coli*

**Figure 1:**
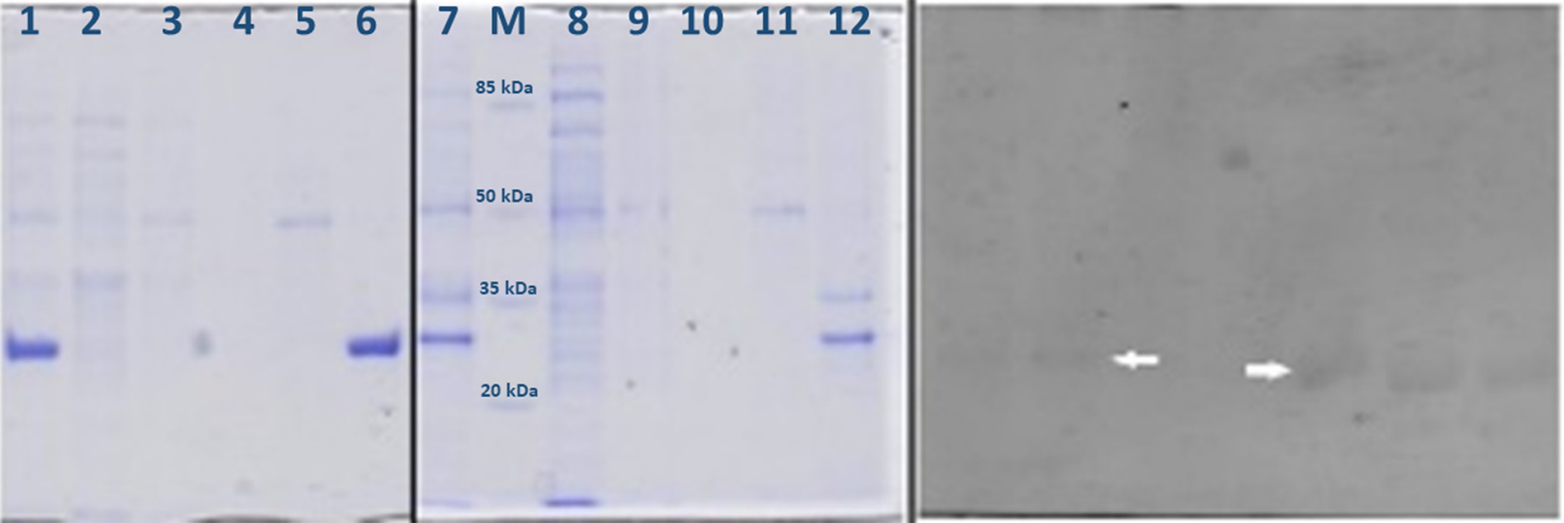
Profile SDS-PAGE Lk2 and Lk3 of the purified protein (**A**) [M] Protein marker III (pre-stained), peqGOLD, [1] Lk3 crude extract, [2] Lk3 flowthrough fraction, [3] Lk3 wash fraction, [4] Lk3 final wash fraction, [5] Lk3 Elution fraction (10 mM imidazole), [6] Lk3 Elution fraction (100 mM imidazole), [7] Lk2 crude extract, [8] Lk2 flowthrough fraction, [9] Lk2 wash fraction, [10] Lk2 final wash fraction, [11] Lk2 Elution fraction (10 mM imidazole), [12] Lk2 Elution fraction (100 mM imidazole). (**B**) Zymogram of Lk2 [1] and Lk3 [2]; (arrow) bands of active band proteins.

Heterologous expression of lipase *LK*2 and *LK*3 genes were carried out by induction with 1 mM IPTG. The proteins were fused with his tag to assist for purification. Cells were lysed by thermolysis method following addition of 0.1% SDS to obtain soluble protein. Following SDS-PAGE analysis, Lk2 and Lk3 were expressed at size around 32 and 31 kDa, respectively (Figure 1A). The proteins still showed lipolytic activity following zymography using naphtyl laurate as substrate (Figure 1B).

IMAC Ni NTA purifications was used to purify the proteins. The purified proteins still shows lipolytic activity. The specific activity of purified Lk2 and Lk3 increased 13 and 12 times compared with that of the crude extracts, respectively (Table 1). Lk2 exhibit higher activity compared with that of Lk3.

**Table 1:** Transesterification activity of crude extract and purified of Lk2 and Lk3

	Total protein (mg)	Total activity (U)	Spesific activity (U/mg)	Purification fold	Yield (%)
C E Lk2	12.55	19.54	1.56	1	100
Purified Lk2	0.41	7.72	20.64	13.25	39.50
C E Lk3	14.47	20.15	1.48	1	100
Purified Lk3	0.82	14.33	17.50	11.85	71.13

U = Unit activity was defined as 1 μmol pNP decreased during reaction per min at 50°C. Methyl palmitate and p-nitrophenol was used as substrates in acetone.

CE = crude extract.

### Lk2 and Lk3 substrate preference

**Figure 2:**
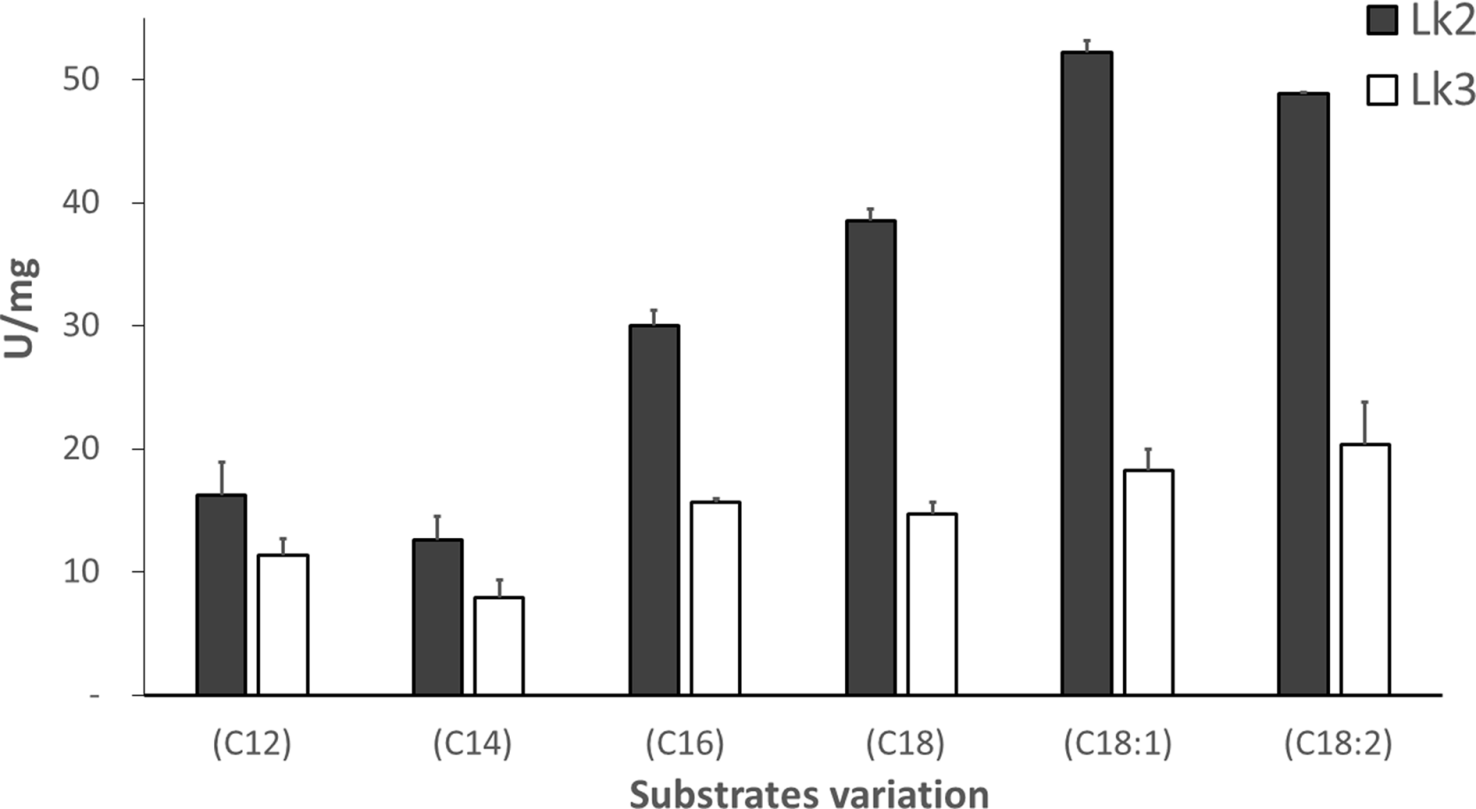
Specific activity of purified protein on variation of long carbon chain substrates (C12) denotes methyl laurate, (C14) methyl myristate, (C16) methyl palmitate, (C18) denotes methyl stearate, (C18:1) denotes methyl oleate, and (C18:2) denotes methyl linoleate.

Lipases are enzymes that are unique to a certain sort of substrate, such as carbon length [38] or a substrate with double bonds in a specific location [39]. The oxyanion hole of *P. stutzeri* lipase (Lk4) was mutated at H110F, which resulted in a shift toward a substrate with a longer carbon chain [40]. Lk2 and Lk3 activity’s were investigated on various methyl ester (C12-C18) and C18 containing double bonds (C18:1, C18:2). The results showed that Lk2 activity was higher than Lk3 activity in each of the substrates tested (C12-C18, C18:1, C18:2). Furthermore, Lk2 preferred C18:1 methyl oleate and Lk3 has similar preference for a range of substrates (Figure 2).

**Figure 3:**
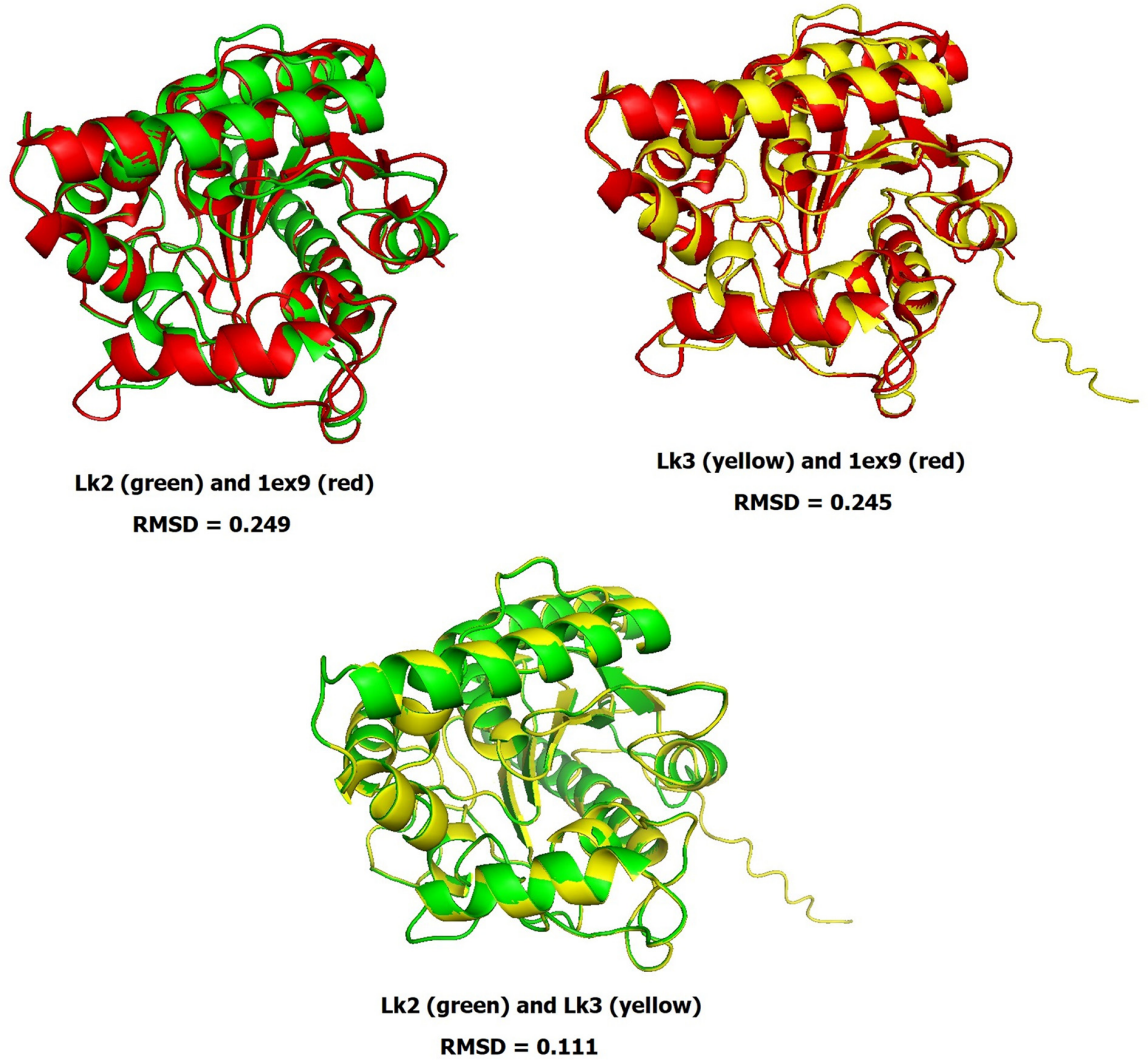
Structure similarity of the *Pseudomonas aeruginosa* lipase (1ex9) with Lk2 and Lk3 These structures generated and aligned by Pymol software.

Lk2 and Lk3 are high homology with percent identical at 89%. Characterization of the structural models by alphafold2 showed that the closest homology to both lipases was the open lid structure of *P. aeruginosa* lipase with PDB ID: 1ex9 (Figure 3).

Both enzymes contains same catalytic triad, however, the geometry and catalytic pocket might different. Although the architecture of the catalytic triad is substantially conserved, the great diversity in the catalytic pocket region may result in varied substrate specificity [41].

**Figure 4:**
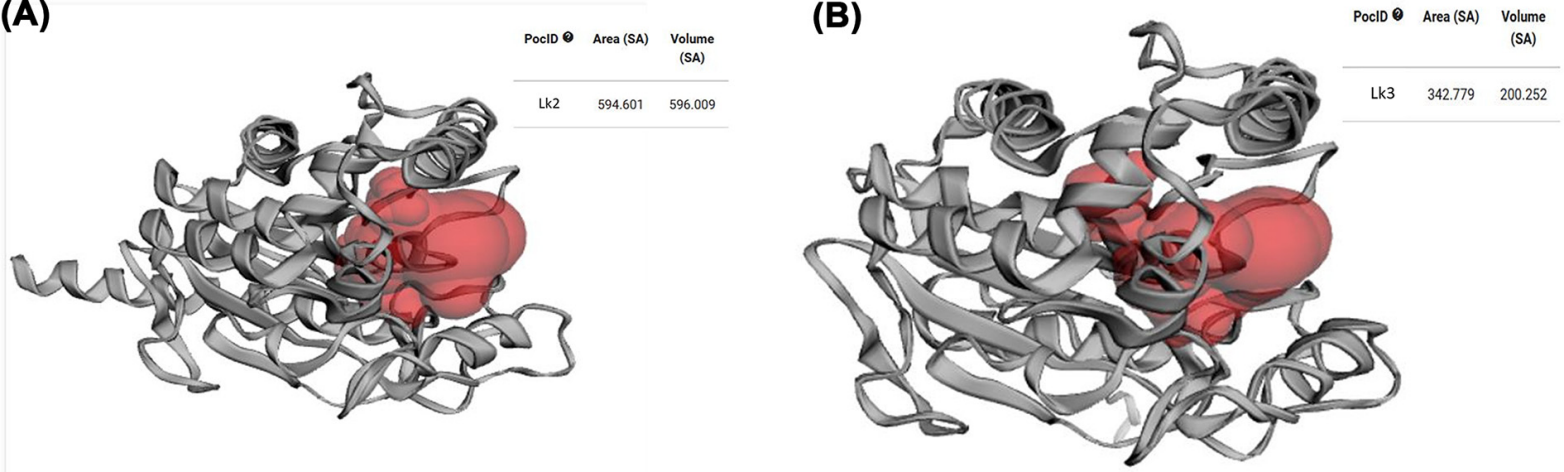
Binding pockets and cavities (**A**) Lk2. (**B**) Lk3 generated by CASTp 3.0 program.

Further characterization based on computational analysis showed that catalytic pocket volume of Lk2 is larger compared with that of Lk3 (Figure 4). This might due to the activity of Lk2 is higher compared with that of Lk3.

On longer carbon chain of substrated up to C18, the activity of the enzyme tends to be more higher compared with that of Lk3. Moreover docking analysis result appeared in agreement with experimental data (Table 2).

**Table 2:** Docking parameters of Lk2 and Lk3 with variation of carbon-length of substrate

		Affinity^1^ (kcal/mol)	Hydrophobic interaction^2^	Hydrogen bond^3^	Salt bridge^4^
				Residue involved	Residue involved
Methyl laurate (12:0)	Lk2	−5.3	8	M19 and S85	H253
	Lk3	--4.6	8	S78	H246
Methyl miristate (14:0)	Lk2	−5.2	4	M19 and S85	H253
	Lk3	--4.7	7	–	H246
Methyl palmitate (16:0)	Lk2	−5.3	5	M19 and S85	H253
	Lk3	--4.8	8	S78	H246
Methyl stearate (18:0)	Lk2	−5.4	8	S85	H253
	Lk3	--5.3	6	S78	H246
Methyl oleate (18:1)	Lk2	−5.7	8	M19 and S85	H253
	Lk3	--5.1	7	–	H246
Methyl linoleate (18:2)	Lk2	−5.7	8	S85	H253
	Lk3	--5.1	8	S78	H246

1 Affinity energy was calculated by Autodock Vina.

^2,3,4^Substrate-enzyme interaction was generated by PLIP program.

M^19^ is one of oxyanion hole of Lk2; S^85^ and H^253^ are catalytic residues of Lk2. S^78^ and H^246^ are catalytic residues of Lk3.

**Figure 5:**
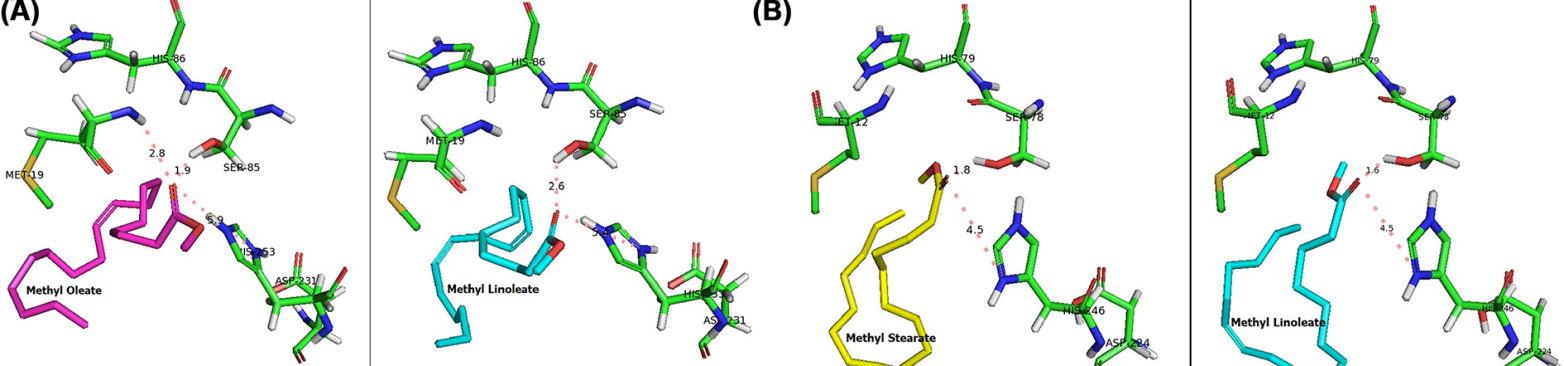
Enzyme–substrate interaction (**A**) Lk2-methyl oleate (purple) and methyl linoleate (blue). (**B**) Lk3-methyl linoleate (blue) and methyl stearate (yellow). Interaction distance in Angstrom (Å). Autodock Vina was used to dock the enzyme–substrate coordinates generated by the Pymol program.

Based on affinity energy and hydrogen bond interaction between substrate and the enzymes showed that Lk2 appeared to have the best interaction with methyl oleate (C18:1) as ligand. Eventhough, the affinity energy of the C18:1 is slightly lower compared with that of C18:2, hydrogen interaction occurred with Met^19^ and S^85^ on C18:1 while on C18:2, the hydrogen interaction only occurred with S^85^. Met^19^ and S^85^ are known as one of oxyanion hole and catalytic triad on Lk2, respectively [19]. Comparison between interaction of C18:1 and C18:2 to Lk2 appeared that methyl oleate interacted with Met^19^ and Ser^85^, while methyl linoleate only interacted with Ser^85^ (Table 2). The interaction distance between methyl oleate and Ser^85^ is longer compared with that of methyl linoleate to Ser^85^ (Figure 5A). Meanwhile for Lk3, C18:2 was appeared as best ligand (Table 2). Eventhough, the affinity energy of C18 is slightly higher compared with that of C18:2; however, hydrogen bond distance between C18:2 to Ser^78^ (0.16 Å) is shorter compared with that of the C18 to Ser^78^ (0.18 Å) (Figure 5B). Ser^78^ is one of catalytic triad on Lk3. Close orientation of catalytic triad to the substrate might cause better interaction and hence increase the activity. All of the computational results are in agreement with the experiment.

### The influence of organic solvents on Lk2 and Lk3 activity

**Figure 6:**
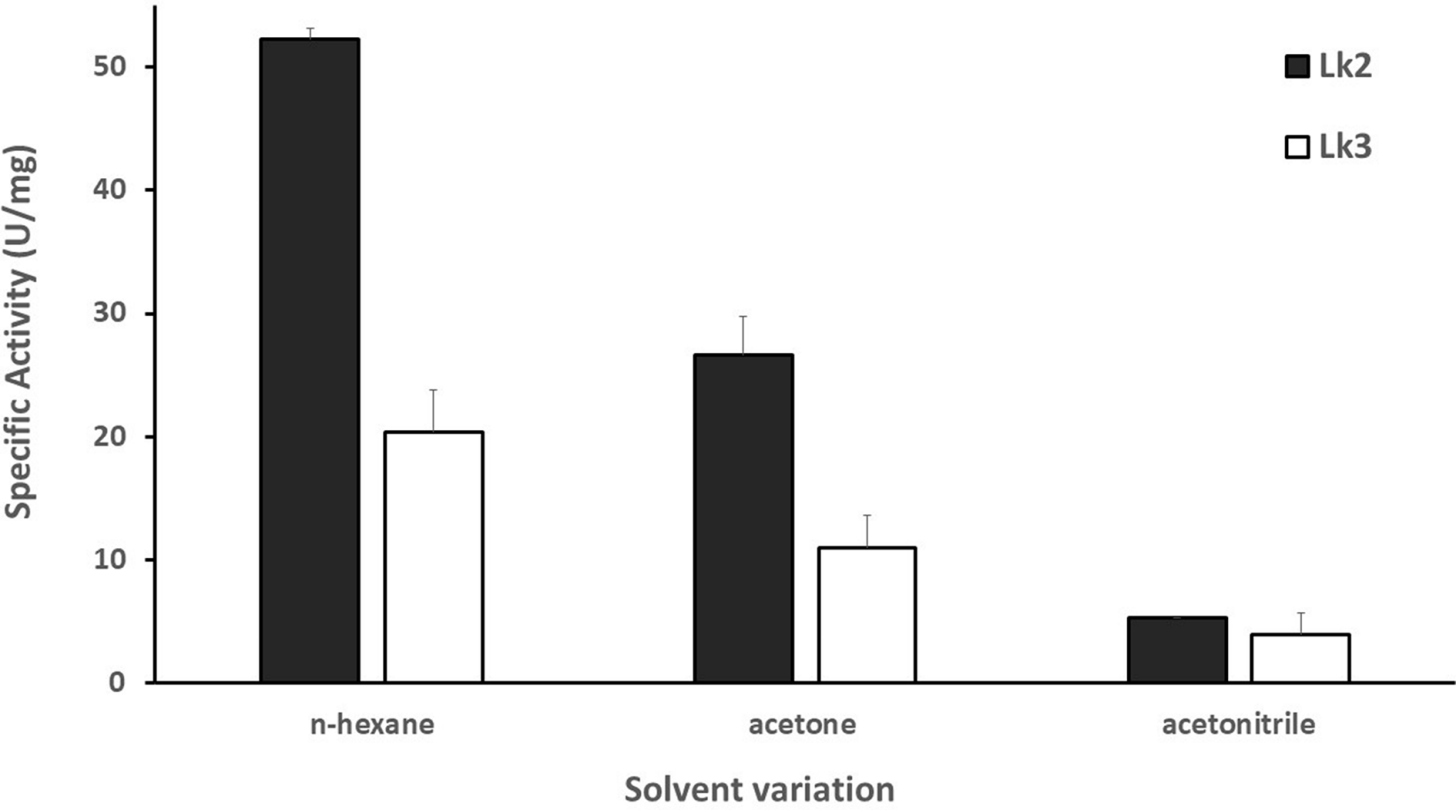
Activity of enzyme on variation of organic solvents

Some lipases’ activity is known to be stable in organic solvents [42]. The transesterification activity of Lk2 and Lk3 was determined using an organic solvent variant. On nonpolar liquids, both enzymes were more active (Figure 6).

**Figure 7:**
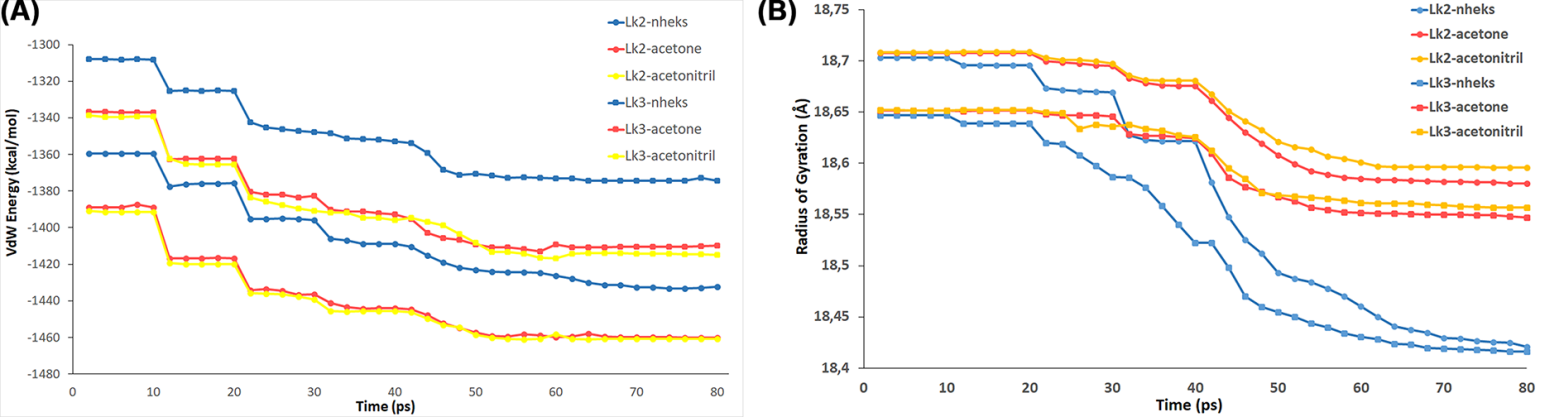
Molecular dynamics simulations based on the NAMD program (**A**) Van Der Waals energy. (**B**) Radius of gyration of Lk2 and Lk3 on various organic solvents.

The highest activity was on n-hexane. The relative activity was decreased up to 80% on acetonitrile compared with that of n-hexane, moreover on acetone the enzymes exhibited 50% activity. Similar result was reported for *Thermomyces lanuginosus* lipase [43]. Some lipases were reported to lose its activity on acetone [21]. The stability of some enzymes on organic solvent was considered to have positive correlation with thermal stability [44]. At high temperatures, the structure of lipase becomes more compact and is retained on native folding in non-polar solvents [45]. Study on the lid movements of *P. stutzeri* lipase (LipC) in water and THF showed that the lid opened wider on THF resulting of catalytic residue of serine exposed on medium [46]. Moreover, molecular dynamics simulation of LipMNK showed unfolded lipase structure on acetonitrile concentration at 80–100% [47]. The highest activity of Lk2 and Lk3 on n-hexane might due to the structure of the enzymes were keep on native folding, while on acetonitrile the structure of enzymes might be unfolded. To probe the above possibilities molecular dynamic simulation was carried out on both Lk2 and Lk3. The result showed that protein–solvent interactions were much stronger on more polar solvents (Figure 7A) in both Lk2 and Lk3 resulting on longer of radius gyration of proteins (Figure 7B).

**Figure 8:**
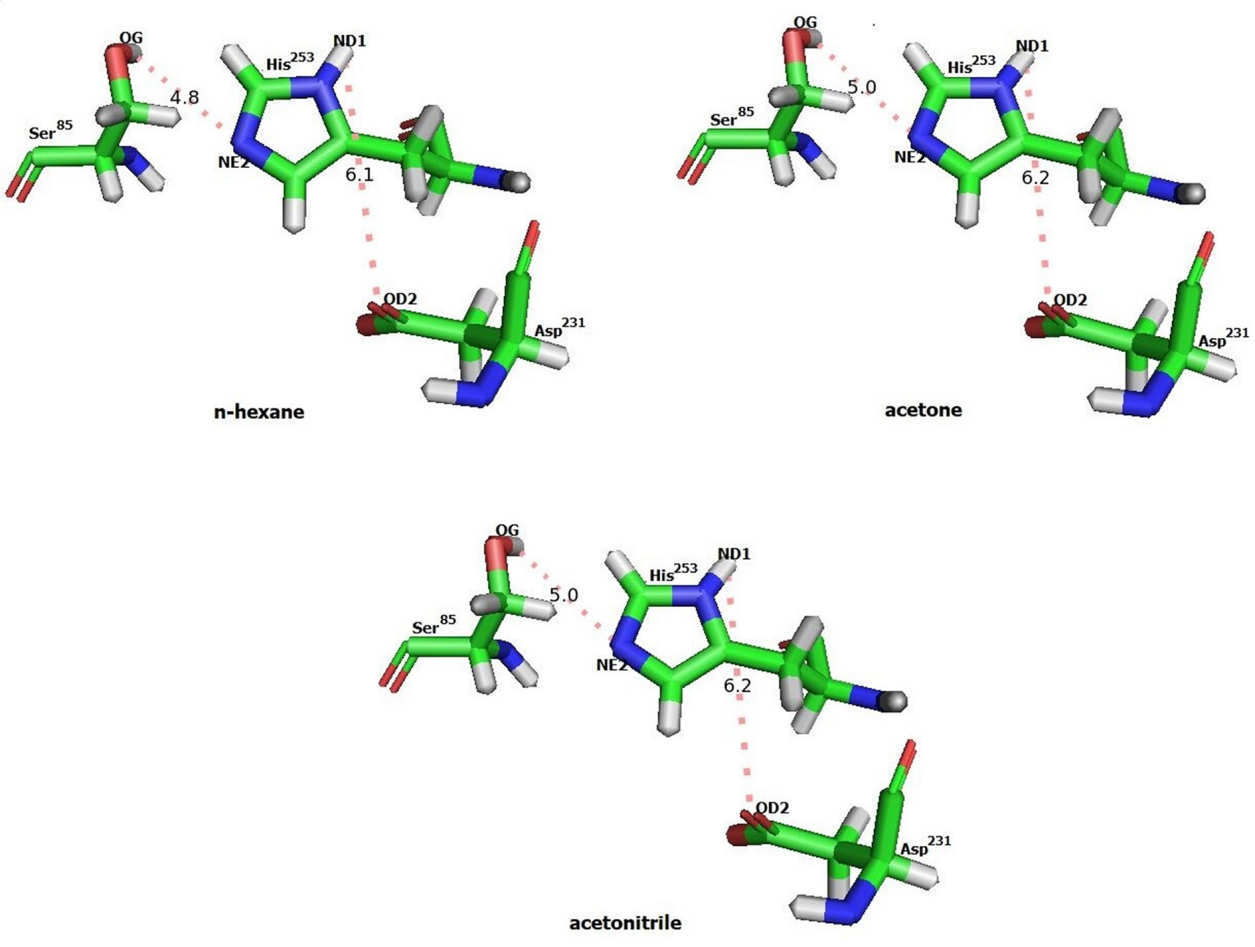
Orientation of triad catalytic residues (Ser, His, Asp) on catalytic center of Lk2 Distance among the residues in Angstrom (Å).

Better interaction of solvent to protein might cause denaturation or conformation change of protein and hence reduce the activity. Previous research showed that the compact 3D structure of proteins in non-polar solvents maintained the conformation of active site [48]. The hydrogen bonding between OG (Ser)-NE2 (His) and OD2 (Asp)-ND1 (His), according to the lipase catalytic mechanism, was critical for the transition state’s stabilization [49]. On Lk2, hydrogen bond distance between OG (Ser^85^)-NE2 (His^246^) and OD2 (Asp^231^)-ND1 (His^246^) is closer in n-hexane (4.8 and 6.1 Å) compared with both in acetone and acetonitrile (5 and 6.2 Å) (Figure 8). This suggested that in n-hexane the enzymes maintained the conformation of the catalytic center as native structure.

### Optimum temperature and thermal stability of Lk2 and Lk3

**Figure 9:**
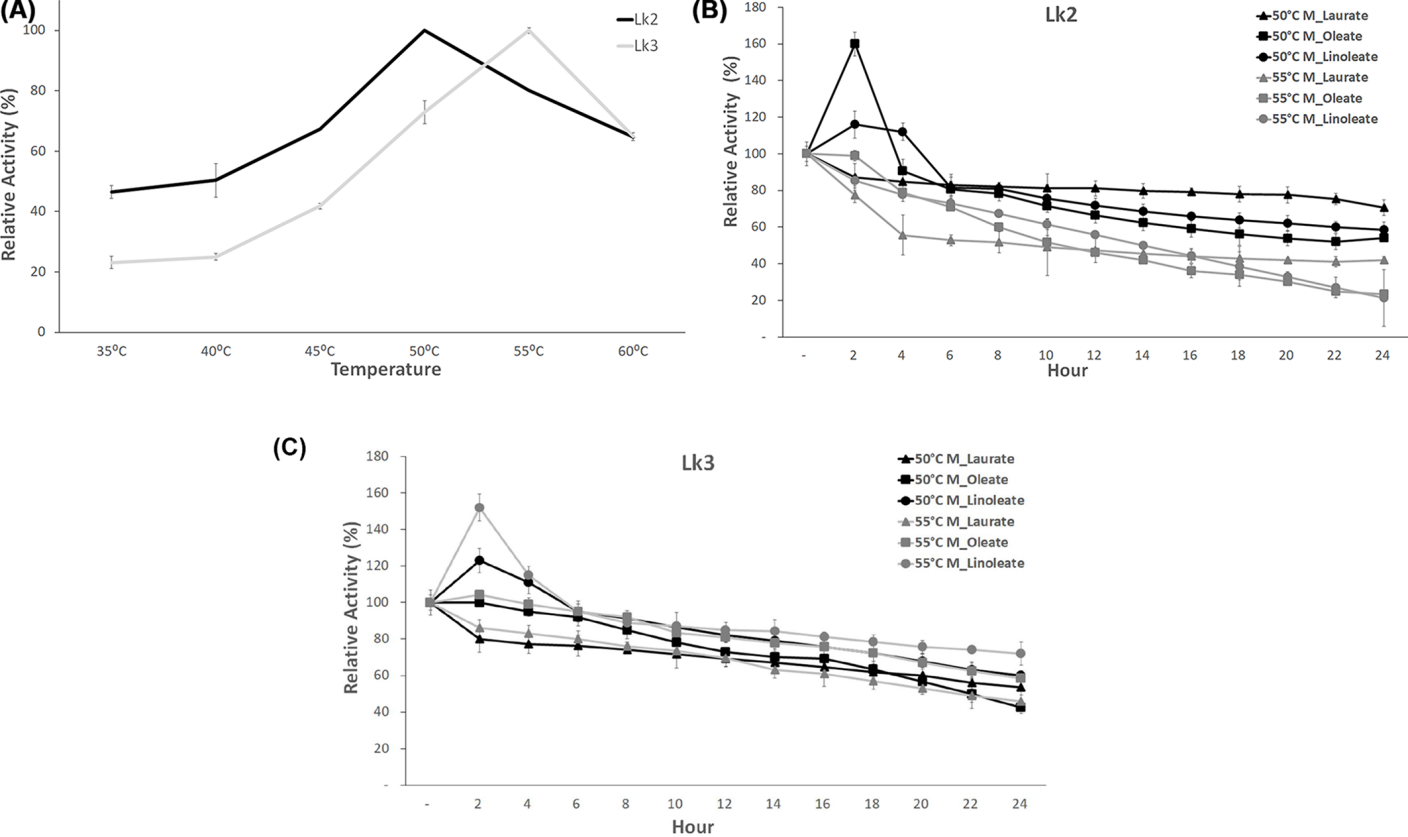
Optimum temperature and thermostability (**A**) Activity of enzyme on variation temperature. (**B**) Thermostability of Lk2 on variation of time incubation. (**C**) Thermostability of Lk3 on variation of time incubation.

Optimum temperature of Lk2 and Lk3 were assayed using methyl palmitate as substrate. The reactions were measured at variation of temperature range from 35 to 60°C. Lk2 and Lk3 showed temperature optimum at 50 and 55°C, respectively (Figure 9A). Most of lipases from thermophilic or thermotolerant bacteria appeared optimum lipolytic activity range at 50–70°C [15,50,51].

Lk2 and Lk3 were tested for thermostability at 50 and 55°C, respectively. The reaction was carried out using n-hexane with three different substrates (methyl laurate, methyl oleate and methyl linoleate). After 2 h of incubation at 50°C, Lk2 activity increased by 1.2- and 1.6-fold each with methyl linoleate and methyl oleate. There is no increasing activity when Lk2 incubated at 55°C. After 24 h of incubation at 50 and 55°C, the highest Lk2 activity was observed on the methyl laurate substrate in comparison with methyl oleate and methyl linoleate (Figure 9B). Meanwhile, after 2 h incubation at 50 and 55°C, Lk3 activity increased 1.2- and 1.6-fold with methyl linoleate. After a 24 h incubation period, the highest activity of Lk3 remained approximately 80% with methyl linoleate (Figure 9C).

Further characterization on thermal stability of the enzymes based on thermal melting prediction analysis [35] showed that Lk3 exhibited a higher *T*_m_ value (72.6°C) compared with that of Lk2 (70.6°C) (Table 3).

**Table 3:** Folding parameter of Lk2 and Lk3 calculated by SCooP program

	Δ*H*_m_ (kcal/mol)	Δ*C*_p_ (kcal/mol K)	*T*_m_ (°C)	ΔG_r_ (kcal/mol)
Lk2	−163.4	−2.81	70.6	--12.8
Lk3	--166.6	--2.25	72.6	−15.2

*T*_m_* =* melting temperature.

Δ*H*_m_* =* the standard folding enthalpy measured at *T*_m._

Δ*C*_p_* =* the standard folding heat capacity.

Δ*^G^*r = folding free energy value at room temperature.

There are many factors influenced on thermostability of protein such as hydrophobic cluster, salt bridge and hydrogen interactions [52–54]. Structure prediction analysis of Lk2 and Lk3 showed that Lk3 contains more hydrophobic cluster and hydrogen bond interaction compared with that of Lk2, respectively (Table 4). All of the *in silico* data is agreement with the experiment that Lk3 is more thermostable compared with that of Lk2.

**Table 4:** Intramolecular interaction of Lk2 and Lk3 generated by Protein Tools Server

	Number of hydrophobic cluster	Number of hydrogen bond	Number of salt bridges
Lk2	5	59	8
Lk3	7	65	8

### Kinetics constants

The constant Michaelis (*K*_m_) and the *V*_max_ were determined from the Lineweaver–Burk double reciprocal plot. These transesterification kinetics were bisubstrate between methyl oleate and p-nitrophenol and also methyl linoleate and p-nitrophenol (Table 5).

**Table 5:** The kinetics parameter of transesterification activity of Lk2 and Lk3

Lipase	Substrates	*V*_max_ (U/mg)	KM ester (mM)	Kcat s^−1^
Lk2	Methyl oleate and pNP	148.38	10.29	81.61
	Methyl linoleate and pNP	114.99	40.56	63.25
Lk3	Methyl oleate and pNP	36.09	13.42	18.65
	Methyl linoleate and pNP	43.79	11.11	22.62

**Figure 10:**
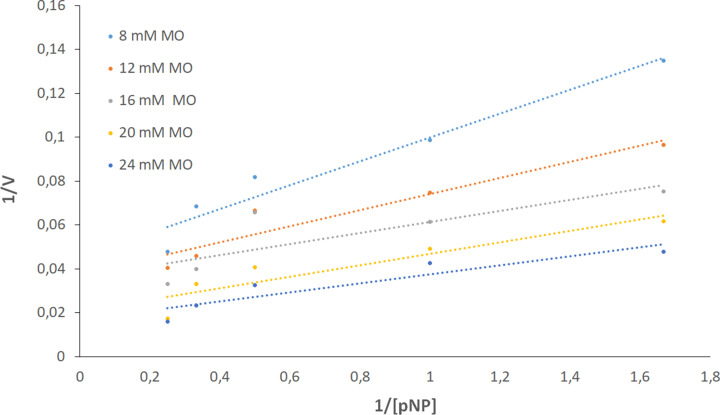
Lineweaver−Burk double-reciprocal plots Dependence of reaction rate as a function of p-nitrophenol concentration at each fixed methyl oleate concentration using 0.48 mg/ml Lk2 concentration at 50°C in n-hexane.

Kinetics mechanism for Lk2 and Lk3 that were categorized as ping-pong double displacement was showed from the plot in Figure 10. Ping-pong (or double displacement) reactions have been highlighted by the formation of parallel lines on a double reciprocal plot [55,56].

The *K*m values for the *Bulkholderia cepacia* lipase were 580 mM with triolein and ethanol as substrates. Commercial lipase Novozyme 435 displayed *K*_m_ values of 29 mM with waste cooking oil and ethanol as substrates. The *K*_m_ and *V*_max_ values for *Candida antartica* lipase A using soybean oil and methanol as substrates were 481 mM and 68.5 U/min. All these enzymes also have ping-pong Bi–Bi mechanisms as kinetics mechanism [57–59].

### Metal ions’ effect on Lk2 and Lk3 activity

**Figure 11:**
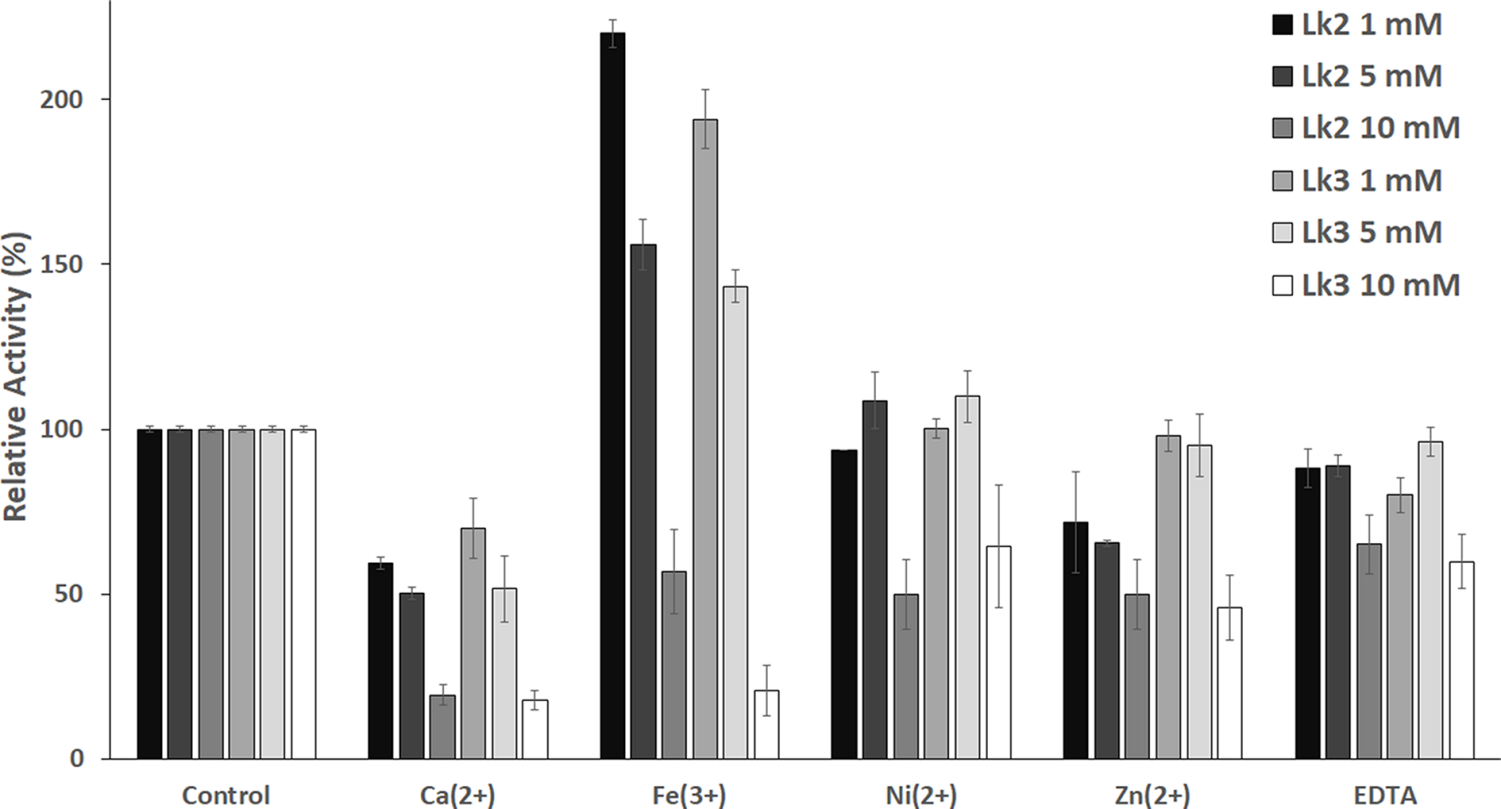
Activity of enzyme on various different metal ions Preincubation of enzymes in 50 mM PBS buffer (pH 8) containing 1, 5 or 10 mM metal ion or EDTA for 30 min at 37°C was used to assess activity. Control without addition of metal ion.

The activity of most enzyme is influenced by the present of metal ion, some of the ions increase the activity [60], some of them inhibit the activity [61]. Lipase family I.1 are known as Ca^2+^ dependent enzyme [3], the presence of Ca^2+^ enhanced the enzyme’s activity considerably. The enzymes contain a conserved region to bind Ca^2+^. In the presence of a variety of metal ions, the enzymes were studied to see how they affected Lk2 and Lk3 activity. Ca^2+^ and Zn^2+^ were shown to inhibit Lk2, while the present of Fe^3+^ and Ni^2+^ increased the activity of the enzyme (Figure 11). The same effect was also exhibited on the activity of Lk3 except for the present of Zn^2+^. The activity of both lipases is slightly affected by EDTA added at a concentration of 1 and 5 mM. Activation and deactivation of both lipases were dose dependent. All metal ion variations and EDTA inhibited lipase activity at a concentration of 10 mM. Increasing the activity of same lipases on the present of Fe^3+^ were reported on *Geobacillus sp*. TW1 lipase [62] and lipase from *P. aeruginosa* [63]. Previous report proposes that redox-inert metal ions tend to stabilize negative charge on the enzyme and activating substrate, while the redox active ions might play a role as Lewis acids and as redox centers [64].

**Figure 12:**
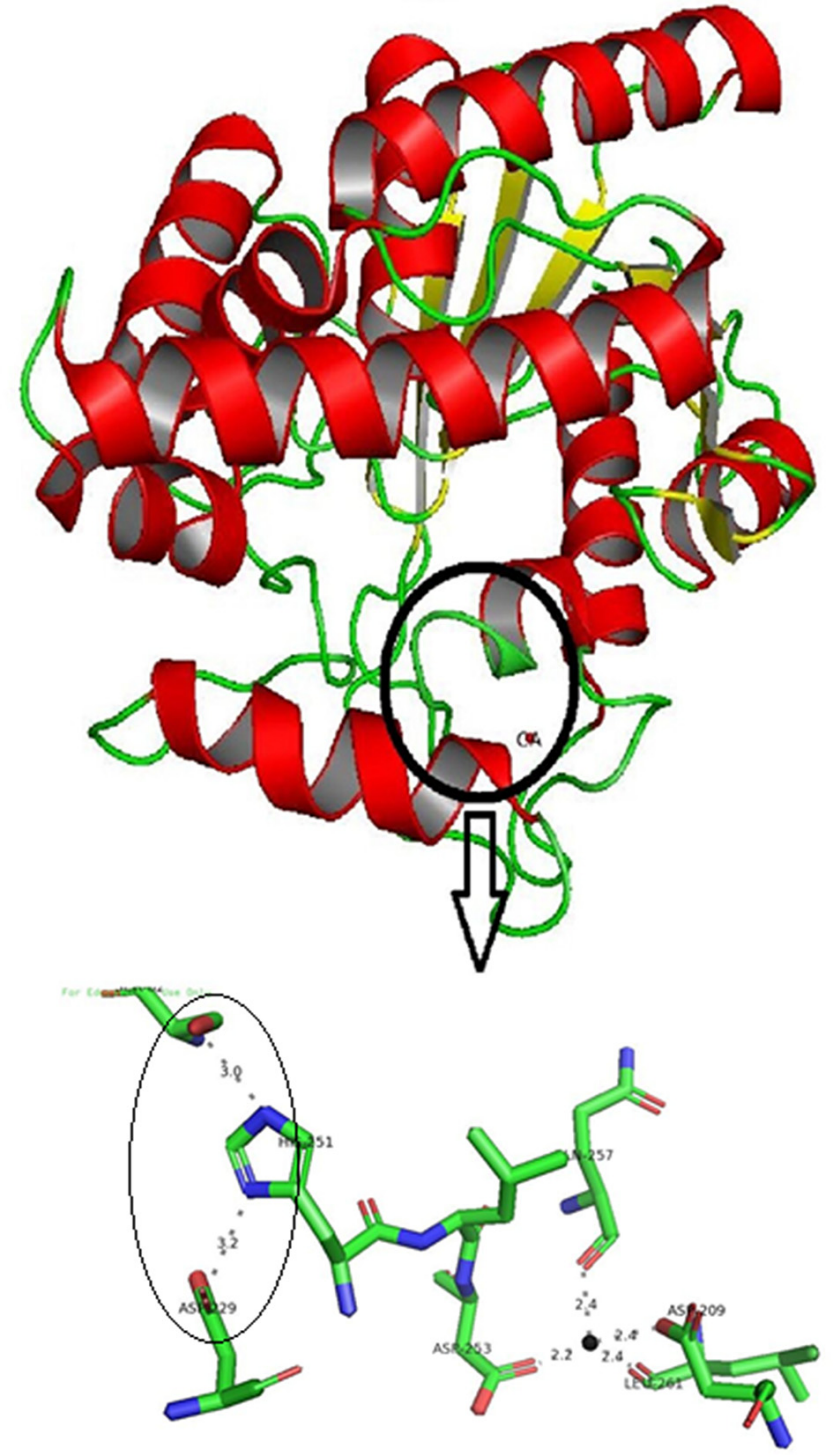
Calcium binding residues and catalytic triad of *P. aeruginosa* lipase (model enzyme for Family I.1 lipase) generated by Pymol program Black circle is Histidine loop area. Distance among the residues in Angstrom (Å). (○) catalytic triad orientations; (●) for Ca^2+^.

**Figure 13:**
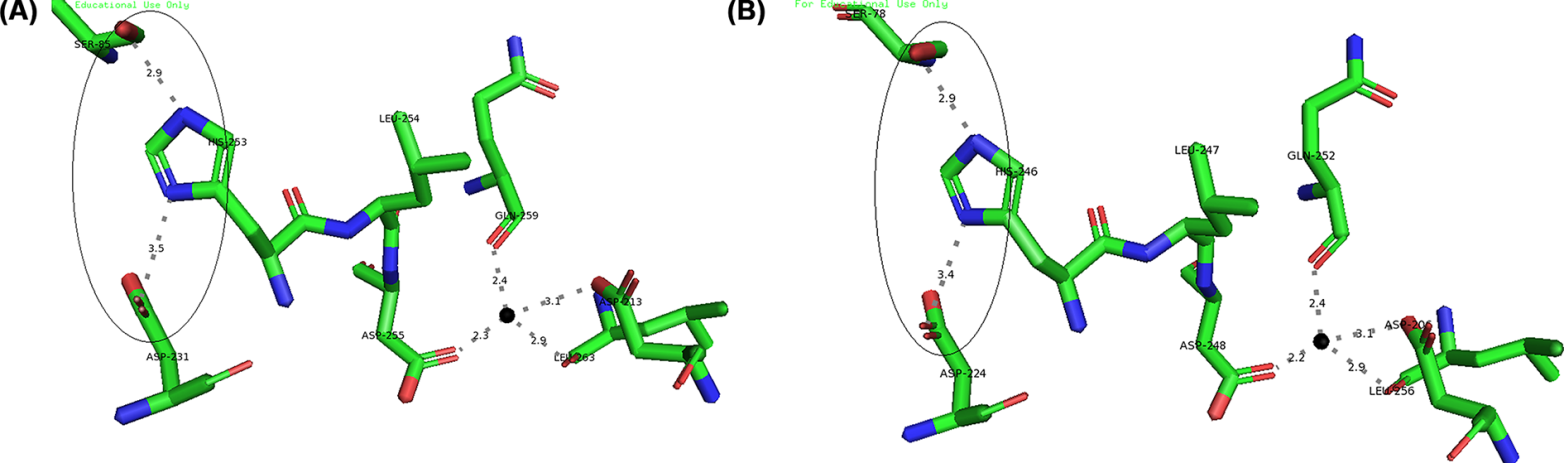
Calcium binding orientation (**A**) Lk2. (**B**) Lk3 generated by MIB Server and visualized by Pymol program. (○) catalytic triad orientations; (●) for Ca^2+^.

Deactivating of Lk2 and Lk3 activities in the present of Ca^2+^ were surprising since Lk2 and Lk3 were belonged to Family I.1 known as Ca^2+^-dependent enzyme. In the present of Ca^2+^, interaction between Ca^2+^ and amino acid residues close to histidine (one of triad catalytic) contributed to keep His at proper orientation in the catalytic center (His loop) and hence activating the enzyme [65]. Conformation or orientation of triad catalytic residues on active center of *P. aeruginosa* lipase was analysed. *Pseudomonas aeruginosa* lipase was used as model enzyme from family I.1 lipase. The result showed that the distance of Ser^85^-His^251^ and His^251^-Asp^229^ are 2.3 and 3.2, respectively. Moreover, the distance between Ca^2+^-amino acid residues is within 2.2 and 2.4 Å (Figure 12). Analysis on the interaction between Ca^2+^-amino acid residues on Lk2 and Lk3 showed that the interaction distance was longer compared with that of interaction on *P. aeruginosa* lipase (Figure 12). The phenomenon affected orientation of Histidine on active center becoming improper (Figure 13). The distance between Ser^85^-His^253^ and His^253^-Asp^231^ in Lk2 were 4.2 and 3.9 Å, respectively. Moreover the distance between Ser^78^-His^246^ and His^246^-Asp^224^ on Lk3 were 3.0 and 4.5 Å, respectively. Orientation shift on catalytic residue of Lk2 and Lk3 caused dramatically decreased on the activity of Lk2 and Lk3. Several previous studies of lipase from metagenomics isolation appeared to have different properties from their related family [66–69]. The above data suggested that Lk2 and Lk3 are unique or novel lipase on family I.1.

## Conclusion

Transesterification activity of Lk2 and Lk3 on various organic solvents was successfully characterized. Lk2 exhibited higher activity compared with that of Lk3 in various carbon length, C12-C18, C18:1 and C18:2. 3D structure prediction of Lk2 contained larger catalytic pocket compared with that of Lk3. Most preference substrate of Lk2 was methyl oleate (C18:1), while for Lk3, the highest activity was appeared on methyl linoleate (C18:2). The preference of substrate on Lk2 and Lk3 were confirmed by computational analysis. The activity of both Lk2 and Lk3 was preferentially increased when a nonpolar organic solvent was present. The enzyme–solvent interaction in n-hexane was weaker than in acetone or acetonitrile. Lk2 was less thermostable compared with that of Lk3. The folding parameter of Lk3 showed that the enzyme is more compact. The activity of both enzymes were activated by the present of Fe^3+^; however, in the present of Ca^2+^ the activity were inhibited. This is contradictive for lipase from family I.1, known as Ca^2+^ dependent enzyme, suggesting that Lk2 and Lk3 are novel lipase on family I.1.

## Data Availability

Lipase gene LK2 and LK3 available in GenBank (https://www.ncbi.nlm.nih.gov/) with accession number of KP204884 and KP204885. All data generated or analyzed during the present study are included in this article and supplementary file.

## Abbreviations

EDTA: ethylenediaminetetraacetate
IPTG: isopropyl β-D-1-thiogalactopyranoside
NAMD: Not (just) Another Molecular Dynamics
PAGE: polyacrylamide gel electrophoresis
PBS: phosphate-buffered saline
SDS: sodium dodecyl sulfate
